# Asymptomatic MERS-CoV Infection in Humans Possibly Linked to Infected Dromedaries Imported from Oman to United Arab Emirates, May 2015

**DOI:** 10.3201/eid2112.151132

**Published:** 2015-12

**Authors:** Zulaikha M. Al Hammadi, Daniel K.W. Chu, Yassir M. Eltahir, Farida Al Hosani, Mariam Al Mulla, Wasim Tarnini, Aron J. Hall, Ranawaka A.P.M. Perera, Mohamed M. Abdelkhalek, J.S.M. Peiris, Salama S. Al Muhairi, Leo L.M. Poon

**Affiliations:** Abu Dhabi Food Control Authority, Abu Dhabi, United Arab Emirates (Z.M. Al Hammadi, Y.M. Eltahir, M.M. Abdelkhalek, S.S. Al Muhairi);; The University of Hong Kong, Hong Kong, China (D.K.W. Chu, R.A.P.M. Perera, J.S.M. Peiris, L.L.M. Poon);; Department of Communicable Diseases, Public Health and Research, Health Authority Abu Dhabi, Abu Dhabi (F. Al Hosani, M. Al Mulla, W. Tarnini);; Centers for Disease Control and Prevention, Atlanta, Georgia, USA (A.J. Hall)

**Keywords:** Middle East respiratory syndrome coronavirus, MERS, MERS-COV, coronavirus, zoonoses, transmission, asymptomatic infection, humans, dromedaries, camels, Oman, United Arab Emirates, epidemiology, viruses

## Abstract

In May 2015 in United Arab Emirates, asymptomatic Middle East respiratory syndrome coronavirus infection was identified through active case finding in 2 men with exposure to infected dromedaries. Epidemiologic and virologic findings suggested zoonotic transmission. Genetic sequences for viruses from the men and camels were similar to those for viruses recently detected in other countries.

Middle East respiratory syndrome (MERS) coronavirus (MERS-CoV) was first detected in humans in 2012 (*1*). Before 2015, most human infections occurred on the Arabian Peninsula. However, the recent occurrence of MERS in South Korea indicates that this pathogen can cause major outbreaks in other regions (*2*). Dromedaries are believed to be a source of MERS-CoV (*3*,*4*), but only a few case reports provide virologic and epidemiologic evidence that directly supports zoonotic transmission of the virus from dromedaries to humans (*5*–*7*). We report the detection of epidemiologically linked MERS-CoV infection in 2 men who had direct contact with infected dromedaries (*8*,*9*).

## The Study

A 29-year-old man (contact 1) transported 8 dromedaries from Oman to United Arab Emirates on May 7, 2015 (Table 1). The same day, as part of a national policy for controlling MERS, samples were collected from the dromedaries at a screening center located at the United Arab Emirates border. The samples were tested by reverse transcription PCR (RT-PCR) on May 10 and found to be positive for the MERS-CoV open reading frame (ORF) 1A and upstream of E genes (*10*). This finding led local public health authorities to conduct active surveillance on humans who had contact with the infected dromedaries.

**Table 1:** A chronology of major events in a study of asymptomatic MERS-CoV infection in 2 humans after direct contact with infected dromedary camels imported from Oman to United Arab Emirates, May 2015*

Date, May 2015	Event
7	Contact 1 transported 8 dromedaries from Oman to the United Arab Emirates border. Contact 2 had direct contact with the dromedaries during sampling procedures at the camel screening center at the border. All 8 dromedaries were quarantined until test results were available on May 10.
10	All 8 dromedaries were found to be RT-PCR positive for MERS-CoV and were quarantined in a separate structure located at the same border location. Active surveillance of persons with direct or indirect contact with the infected dromedaries was initiated. A sputum sample was obtained from contact 1; it tested positive for MER-CoV by RT-PCR on 12 May, 2015.
12	A sample obtained from contact 1 on May 10 tested positive for MERS-CoV by RT-PCR; contact 1 was hospitalized in a negative-pressure room.
13	A follow-up sample was obtained from contact 1, and it tested positive for MERS by RT-PCR.†
14	A follow-up sample was obtained from contact 1, and it tested positive for MERS by RT-PCR. A nasal aspirate sample was obtained from contact 2; it tested positive for MERS by RT-PCR on May 17.† Samples were obtained from the infected dromedaries, and 5 were still MERS-CoV–positive by RT-PCR (Table 2).†
17	A sample obtained from contact 2 on May 14 tested positive for MERS-CoV by RT-PCR.
18	Contact 2 was admitted to a negative-pressure room in the same hospital as contact 1. Follow-up samples were obtained from contacts 1 and 2, and they tested negative for MERS-CoV by RT-PCR.
20	A follow-up sample was obtained from contact 2, and it tested negative for MERS-CoV by RT-PCR.
21	A follow-up sample was obtained from contact 2, and it tested negative for MERS-CoV by RT-PCR.
25	Follow-up samples from the 5 dromedaries tested negative for MERS-CoV by RT-PCR. All dromedaries were released from quarantine.
End of month‡	Contacts 1 and 2 were released uneventfully from the hospital.

*Contacts 1 and 2, humans who had direct physical contact with infected dromedaries; MERS-CoV, Middle East respiratory syndrome; RT-PCR, reverse transcription PCR. †Samples subjected to sequencing analyses. ‡Exact date unknown.

A sputum sample collected from contact 1 on May 10, 2015, was tested by RT-PCR on May 12 and found to be positive for MERS-CoV; the man was admitted to a hospital the same day. Follow-up respiratory samples obtained on May 13 and 14 were still RT-PCR–positive, but a sample obtained on May 18 was negative. The patient was asymptomatic at hospital admission and throughout his hospital stay (Technical Appendix).

Contact 2 was a 33-year-old man who worked at the screening center mentioned above. He had direct contact with the same group of infected dromedaries during the sampling procedures. A nasal aspirate sample was obtained from the man on May 14, 2015, and found to be RT-PCR positive for MERS-CoV. Contact 2 was hospitalized on May 18. A follow-up sample obtained on May 18 was RT-PCR negative for MERS-CoV. Contact 2 was asymptomatic throughout his hospitalization (Technical Appendix).

Samples from 32 other persons were also tested by RT-PCR (Technical Appendix). None tested positive.

After the initial positive test results, the dromedaries were quarantined. Seven days later (May 14), follow-up nasal swab samples from 5 dromedaries were still positive by RT-PCR (Table 2); the animals also had mucopurulent nasal discharge. The animals were tested for the presence of MERS-CoV–specific neutralizing antibodies (*11*); all were seropositive. Two 4-month-old calves (ADFCA-HKU1 and ADFCA-HKU2) had the highest virus loads by real-time RT-PCR and the lowest neutralizing antibody titers (Table 2). Nasal swab samples from these 2 dromedaries were also MERS-CoV–positive by rapid antigen testing (*12*), which suggests the calves were still shedding virus 7 days after the first detection of virus. Virus culture was not attempted. On May 25, 2015, the 2 calves were RT-PCR negative for MERS-CoV, and the whole group of camels was released from quarantine.

**Table 2:** Demographic data and clinical test results for MERS-CoV–infected dromedary camels imported from Oman to United Arab Emirates, May 2015*

Camel ID	Age/sex	Purpose of importation	Mucopurulent nasal discharge†	Test results		
				RT-PCR (C_t_)†‡	Rapid antigen test†§	Serum neutralizing antibody titer†¶
ADFCA-HKU1	4 m/F	Breeding	Moderate	Pos (24.54)	Pos	1:40
ADFCA-HKU2	4 m/F	Breeding	Moderate	Pos (27.59)	Pos	1:40
ADFCA-HKU3	4 m/F	Breeding	Moderate	Pos (28.82)	Neg	1:80
ADFCA-HKU4	7 m/F	Breeding	Moderate	Pos (29.81)	Neg	1:80
ADFCA-HKU5	10 y/F	Breeding	Mild	Pos (30.05)	Neg	1:160

*C_t_, cycle threshold; ID, identification; MERS-CoV, Middle East respiratory syndrome; Neg, negative; Pos, positive; RT-PCR, reverse transcription PCR. †Observations or samples from the second sampling on May 14, 2015. ‡Results were determined by open reading frame 1A and upstream of E gene RT-PCR assays; C_t_ values were from the open reading frame 1A assay. C_t_ values from both assays were comparable (data not shown). §Results were determined by immunochromatographic tests for MERS-CoV nucleocapsid protein (*12*). ¶Results were determined by pseudoparticle neutralization assays (*11*).

Respiratory specimens from the 2 infected humans and the 5 dromedaries that were still positive at the second sampling were analyzed by dideoxy sequencing as previously described (*13*); the nucleocapsid gene sequences of all dromedary samples were found to be identical. Samples from dromedaries ADFCA-HKU1–3 were selected for further analysis, and a sequence contig encompassing the 3′ end of the ORF1AB gene through the 3′ untranslated region of the MERS-CoV genome (≈8,900 nt; sequence coverage 4) was obtained from each sample. Contigs from the 3 samples were identical, with the exception of a V221I (GTT→ATT) mutation in the ORF4b protein of the sample from dromedary ADFCA-HKU2. The viral RNA content of the 2 human samples available for analysis was too low to provide long PCR amplicons (cycle threshold 35.5 and 36.9 by upstream of E gene assay). However, partial sequences of MERS-CoV spike (466 nt, contacts 1 and 2), ORF3–4a (273 nt, contact 1), and nucleocapsid (451 nt, contacts 1 and 2) gene regions could be detected from the samples. All of these sequences were identical to those deduced from the dromedary specimens. Genomic sequences determined from this study were submitted to GenBank (accession nos. KT275306–KT275315).

The 3 sequence contigs obtained from the dromedary samples were phylogenetically closely related to those of viruses detected in humans in the Saudi Arabia, China, and South Korea in 2015 (Figure). All sequences from this cluster, together with the partial ORF3–4a sequence detected in the sample from contact 1, shared 2 cluster-specific mutations, 79S (TCA→TCT) and P86L (CCT→ CTT), in the ORF3 protein, suggesting that these viruses may share a common lineage. Apart from the unique V221I mutation, the sequences for viruses from the 3 dromedaries shared a unique ORF4a-Q102E (GAG→CAG) mutation that was not found in any published MERS-CoV genomes. Other than those mutations, all of the ORFs (nonstructural protein 13, spike, ORF3, ORF5, envelope, membrane, nucleocapsid, and ORF8b) of these virus sequences were unremarkable.

**Figure:**
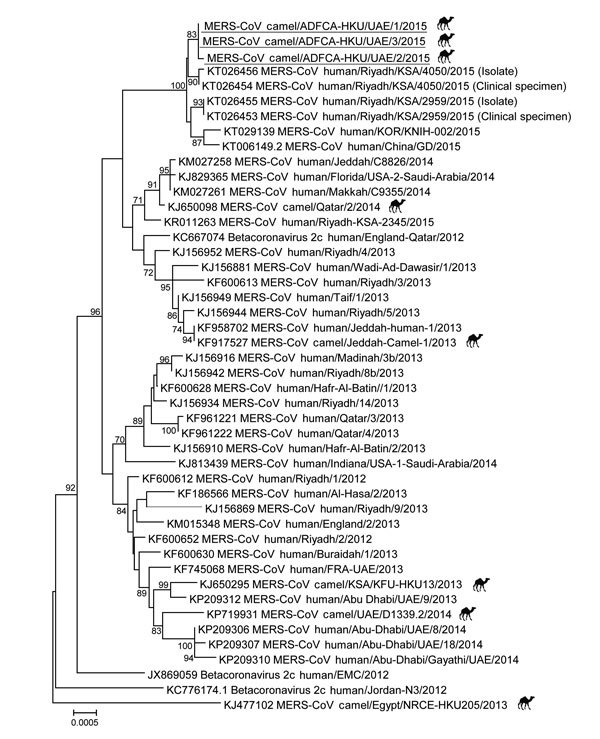
Phylogenetic analyses of partial Middle East respiratory syndrome coronavirus (MERS-CoV) genomic sequences for viruses detected in dromedaries imported from Oman to United Arab Emirates, May 2015. A partial viral RNA sequence spanning the 3′ end of the open reading frame 1AB gene through the 3′ untranslated region of the MERS-CoV genome (≈8,900 nt) was used in the analysis. The phylogenetic tree was constructed with MEGA6 software (http://www.megasoftware.net/) by using the neighbor-joining method. Numbers at nodes indicate bootstrap values determined by 1,000 replicates. Only bootstrap values >70 are denoted. Underlining indicates sequences for viruses detected in this study. GenBank accession numbers are shown for published sequences. Symbols indicate MERS-CoVs detected from dromedaries s. Scale bar indicates the estimated genetic distance of these viruses.

## Conclusions

We report 2 cases of MERS-CoV infection in men who had direct contact with the same group of infected dromedaries. Neither man had a concurrent medical condition or a history of exposure to human MERS cases in the 14 days before their first MERS-CoV–positive test results. Genomic sequences for the viruses derived from the men and dromedaries and findings from the epidemiologic investigation suggest possible zoonotic transmission of MERS-CoV from dromedaries to humans. Although it is unlikely, we cannot exclude the possibility that the men and dromedaries were independently infected by other sources.

Both infected humans were kept in the hospital for ≈2 incubation periods and were asymptomatic during this period. Clinical observations and positive RT-PCR results suggest that the men were asymptomatically infected with MERS-CoV. Asymptomatic infections have been detected previously (*14*). Our findings provide further evidence that asymptomatic human infections can be caused by zoonotic transmission. It is not clear whether asymptomatic infection can lead to transmission between humans. Nonetheless, our findings highlight the importance of systematic surveillance of persons who have frequent contact with dromedaries. A recent study demonstrated that persons who have frequent exposure to camels are more likely than the general population to be seropositive for MERS-CoV (*4*). The unique border screening program and multisectoral collaborations highlighted in this investigation serve as a model for effective MERS-CoV surveillance at the animal–human interface.

Our study had some limitations. We did not test serum samples from the human contacts; such testing would be of interest for follow-up investigation of the patients’ serologic responses. We also obtained limited RNA samples from these persons, which prevented us from conducting more extensive viral sequence analyses.

MERS-CoV genomic sequences determined in this study are similar to those of viruses detected in 2015 in patients in Saudi Arabia and South Korea with hospital-acquired infections. The infected dromedaries in this study were imported from Oman, which suggests that viruses from this clade are widely circulating on the Arabian Peninsula. Sequence analyses of MERS-CoVs found in South Korea and China do not suggest that viruses from this clade are necessarily more transmissible variants (*15*). However, given that a single introduction of MERS-CoV from this clade caused >180 human infections in hospital settings (*2*) and that viruses of this clade are causing other human infections in Saudi Arabia, further phenotypic risk assessment of this particular MERS-CoV clade should be a priority.

Technical Appendix Additional information regarding 2 persons with asymptomatic MERS-CoV infection and other persons tested in the study.

## References

[1] Zaki AM, van Boheemen S, Bestebroer TM, Osterhaus AD, Fouchier RA. Isolation of a novel coronavirus from a man with pneumonia in Saudi Arabia. N Engl J Med. 2012;367:1814–20 . 10.1056/NEJMoa1211721 23075143

[2] World Health Organization. Middle East respiratory syndrome coronavirus (MERS-CoV): summary and risk assessment of current situation in the Republic of Korea and China—as of 19 June 2015 [cited 2015 Aug 8]. http://www.who.int/emergencies/mers-cov/mers-cov-republic-of-korea-and-china-risk-assessment-19-june-2015.pdf?ua=1

[3] Zumla A, Hui DS, Perlman S. Middle East respiratory syndrome. Lancet. 2015 Jun 3 [Epub ahead of print]. **PMID: 26049252** 10.1016/S0140-6736(15)60454-8 PMC4721578 26049252

[4] Müller MA, Meyer B, Corman VM, Al-Masri M, Turkestani A, Ritz D, Presence of Middle East respiratory syndrome coronavirus antibodies in Saudi Arabia: a nationwide, cross-sectional, serological study. Lancet Infect Dis. 2015;15:559–64. 10.1016/S1473-3099(15)70090-3 25863564 PMC7185864

[5] Azhar EI, El-Kafrawy SA, Farraj SA, Hassan AM, Al-Saeed MS, Hashem AM, Evidence for camel-to-human transmission of MERS coronavirus. N Engl J Med. 2014;370:2499–505. 10.1056/NEJMoa1401505 24896817

[6] Memish ZA, Cotten M, Meyer B, Watson SJ, Alsahafi AJ, Al Rabeeah AA, Human infection with MERS coronavirus after exposure to infected camels, Saudi Arabia, 2013. Emerg Infect Dis. 2014;20:1012–5. 24857749 10.3201/eid2006.140402 PMC4036761

[7] Haagmans BL, Al Dhahiry SH, Reusken CB, Raj VS, Galiano M, Myers R, Middle East respiratory syndrome coronavirus in dromedary camels: an outbreak investigation. Lancet Infect Dis. 2014;14:140–5. 10.1016/S1473-3099(13)70690-X 24355866 PMC7106553

[8] World Health Organization. Emergencies preparedness, response. Middle East respiratory syndrome coronavirus (MERS-CoV)—United Arab Emirates [cited 2015 Jun 29]. http://www.who.int/csr/don/18-may-2015-mers-are/en/

[9] World Health Organization. Global alert and response (GAR). Middle East respiratory syndrome coronavirus (MERS-CoV)—United Arab Emirates [cited 2015 Jun 29]. http://www.who.int/csr/don/24-may-2015-mers-are/en/

[10] Corman VM, Muller MA, Costabel U, Timm J, Binger T, Meyer B, Assays for laboratory confirmation of novel human coronavirus (hCoV-EMC) infections. Euro Surveill. 2012;17:20334 . 23231891 10.2807/ese.17.49.20334-en

[11] Perera RA, Wang P, Gomaa MR, El-Shesheny R, Kandeil A, Bagato O, Seroepidemiology for MERS coronavirus using microneutralisation and pseudoparticle virus neutralisation assays reveal a high prevalence of antibody in dromedary camels in Egypt, June 2013. Euro Surveill. 2013;18:20574 . 24079378 10.2807/1560-7917.es2013.18.36.20574

[12] Song D, Ha G, Serhan W, Eltahir Y, Yusof M, Hashem F, Development and validation of a rapid immunochromatographic assay for detection of Middle East respiratory syndrome coronavirus antigen in dromedary camels. J Clin Microbiol. 2015;53:1178–82. 10.1128/JCM.03096-14 25631809 PMC4365254

[13] Chu DK, Poon LL, Gomaa MM, Shehata MM, Perera RA, Abu Zeid D, MERS coronaviruses in dromedary camels, Egypt. Emerg Infect Dis. 2014;20:1049–53. 10.3201/eid2006.140299 24856660 PMC4036765

[14] Oboho IK, Tomczyk SM, Al-Asmari AM, Banjar AA, Al-Mugti H, Aloraini MS, 2014 MERS-CoV outbreak in Jeddah—a link to health care facilities. N Engl J Med. 2015;372:846–54 . 10.1056/NEJMoa1408636 25714162 PMC5710730

[15] World Health Organization. Emergencies preparedness, response. Middle East respiratory syndrome coronavirus (MERS-CoV)—Republic of Korea [cited 2015 Jun 29]. http://www.who.int/csr/don/12-june-2015-mers-korea/en/

