# Supplementary material for: Asymptomatic MERS-CoV Infection in Humans Possibly Linked to Infected Dromedaries Imported from Oman to United Arab Emirates, May 2015
Source: Emerg Infect Dis. 2015 Dec;21(12):2197–200. doi: 10.3201/eid2112.151132 (PMC4672428; doi:10.3201/eid2112.151132)
Supplement: Technical Appendix — Additional information regarding 2 persons with asymptomatic MERS-CoV infection and other persons tested in the study. [file 15-1132-Techapp-s1.pdf]

Article DOI: http://dx.doi.org/10.3201/eid2112.151132

# Asymptomatic MERS-CoV Infection in Humans Possibly Linked to Infected Camels Imported from Oman to United Arab Emirates, May 2015

## Technical Appendix

### Additional Information[1] Regarding Contacts 1 and 2 and Other Persons Tested in the Study

#### Information Regarding Contact 1

Contact 1 is a truck driver who frequently transports camels from Oman to United Arab Emirates. He confirmed that he had direct contact (i.e. direct physical contact) with the infected camels in this particular transportation. A sputum sample collected on 10 May, 2015, was positive for MERS-CoV by RT-PCR on 12 May, 2015. The man was asymptomatic at the time of sample collection. He was admitted to a negative pressure room in a hospital on 12 May, 2015. At the time of his admission, his temperature was 36.9°C, his heart rate was 78 beats per minute, his blood pressure was 137/54 mmHg, and his respiratory rate was 17 breaths per minute. An aspirate sample was collected on the same day and tested positive for MERS-CoV by PCR on 13 May and he was started on Tamiflu (75 mg bid). The man had no comorbid conditions. He had no history of exposure to MERS patients in the 14 days prior to detection of MERS-CoV. He remained asymptomatic during his hospital stay. He was tested to be negative before leaving the hospital at

the end of May. An official announcement was released by the corresponding health authority (http://www.haad.ae/haad/tabid/58/ctl/Details/Mid/417/ItemID/487/Default.aspx).

**Information Regarding Contact 2**

Contact 2 worked in the border screening center as a cleaner. Because of his job nature, he had frequent direct contact with camels in the center. He was tested twice by RT-PCR assay for MERS-CoV in February 2015 and found to be negative both times. His exposure in May 2015 was considered to be a direct contact (i.e. direct physical contacts). On May 7, 2015, without wearing any personal protective equipment, the man held onto the infected dromedary camels (DCs) while the veterinarian obtained nasal swab samples from the animals..[2]

Nasal aspirate sample taken from Contact 2 on 14 May, 2015, was found to be RT-PCR positive for MERS-CoV. He reported no symptoms at that time. He was admitted to a negative pressure room in a hospital for close monitoring and quarantine. At the time of his admission, his temperature was 36.9°C, his heart rate was 77 beats per minute, his respiratory rate was 16 breaths per minute, and his blood pressure was 132/91 mmHg. The patient did not report any history of comorbid conditions such as cardiovascular diseases, diabetes, chronic pulmonary diseases, rheumatic diseases, liver diseases, renal diseases, or malignant disorders. The patient was interviewed twice for obtaining a detailed history using two different formats. The patient did not report recent history of fever, cough, sore throat, runny nose, earache, chest pain, abdominal pain, body ache, vomiting, diarrhea, headache, or any other complaints. The patient received Tamiflu (75mg bid) starting on May 19, 2015. The patient was not given steroids at that time. The patient did not report any history of recent travel in the 10 days before testing. The patient was interviewed again on May 24, 2015, by an infectious diseases officer using a different form for obtaining additional information related to animal exposure. The patient was interviewed in Arabic. The

patient lived in a compound that is about 10 km from the camel screening center. The patient worked as a cleaner in this center for 20 months (working hours: 72 hours per week). He did not report any history of direct contact with people who had respiratory illness or fever within the 14 days prior to testing. He did not have any recent visits to a hospital. He was tested to be negative before leaving the hospital at the end of May. An official announcement was released by the corresponding health authority (http://www.haad.ae/haad/tabid/58/ctl/Details/Mid/417/ItemID/487/Default.aspx).

### Information Regarding Additional Persons Screened in the Study

An additional 32 persons were tested in the study. One of these persons was confirmed to have direct physical contact with the MERS-CoV–positive DCs. The other 31 persons were found to have close contact with the MERS-CoV–positive DCs or humans. None of them was RT-PCR positive for MERS-CoV.

## Remarks

[1]Some information from Contacts 1 and 2 was obtained using the ISARIC Case Record Form for Cases of Human Infection with the Novel Coronavirus.

[2]Unlike the practice of Contact 2, full PPE is routinely worn by members in the veterinary team who are in charge of taking camel samples in the screening center. None of the members in the veterinary team had positive results for MERS-CoV since the implementation of the borders screening.
